# DDX5 resolves R-loops at DNA double-strand breaks to promote DNA repair and avoid chromosomal deletions

**DOI:** 10.1093/narcan/zcaa028

**Published:** 2020-09-25

**Authors:** Zhenbao Yu, Sofiane Y Mersaoui, Laure Guitton-Sert, Yan Coulombe, Jingwen Song, Jean-Yves Masson, Stéphane Richard

**Affiliations:** Segal Cancer Center, Lady Davis Institute for Medical Research and Gerald Bronfman Department of Oncology and Departments of Biochemistry, Human Genetics and Medicine, McGill University, Montréal, Québec H3T 1E2, Canada; Segal Cancer Center, Lady Davis Institute for Medical Research and Gerald Bronfman Department of Oncology and Departments of Biochemistry, Human Genetics and Medicine, McGill University, Montréal, Québec H3T 1E2, Canada; Genome Stability Laboratory, CHU de Québec Research Center, Oncology Axis, Department of Molecular Biology, Medical Biochemistry and Pathology, Laval University Cancer Research Center, 9 McMahon, Québec City, Québec G1R 3S3, Canada; Genome Stability Laboratory, CHU de Québec Research Center, Oncology Axis, Department of Molecular Biology, Medical Biochemistry and Pathology, Laval University Cancer Research Center, 9 McMahon, Québec City, Québec G1R 3S3, Canada; Genome Stability Laboratory, CHU de Québec Research Center, Oncology Axis, Department of Molecular Biology, Medical Biochemistry and Pathology, Laval University Cancer Research Center, 9 McMahon, Québec City, Québec G1R 3S3, Canada; Segal Cancer Center, Lady Davis Institute for Medical Research and Gerald Bronfman Department of Oncology and Departments of Biochemistry, Human Genetics and Medicine, McGill University, Montréal, Québec H3T 1E2, Canada; Genome Stability Laboratory, CHU de Québec Research Center, Oncology Axis, Department of Molecular Biology, Medical Biochemistry and Pathology, Laval University Cancer Research Center, 9 McMahon, Québec City, Québec G1R 3S3, Canada; Segal Cancer Center, Lady Davis Institute for Medical Research and Gerald Bronfman Department of Oncology and Departments of Biochemistry, Human Genetics and Medicine, McGill University, Montréal, Québec H3T 1E2, Canada

## Abstract

R-loops are three-stranded structures consisting of a DNA/RNA hybrid and a displaced DNA strand. The regulatory factors required to process this fundamental genetic structure near double-strand DNA breaks (DSBs) are not well understood. We previously reported that cellular depletion of the ATP-dependent DEAD box RNA helicase DDX5 increases R-loops genome-wide causing genomic instability. In this study, we define a pivotal role for DDX5 in clearing R-loops at or near DSBs enabling proper DNA repair to avoid aberrations such as chromosomal deletions. Remarkably, using the non-homologous end joining reporter gene (EJ5-GFP), we show that DDX5-deficient U2OS cells exhibited asymmetric end deletions on the side of the DSBs where there is overlap with a transcribed gene. Cross-linking and immunoprecipitation showed that DDX5 bound RNA transcripts near DSBs and required its helicase domain and the presence of DDX5 near DSBs was also shown by chromatin immunoprecipitation. DDX5 was excluded from DSBs in a transcription- and ATM activation-dependent manner. Using DNA/RNA immunoprecipitation, we show DDX5-deficient cells had increased R-loops near DSBs. Finally, DDX5 deficiency led to delayed exonuclease 1 and replication protein A recruitment to laser irradiation-induced DNA damage sites, resulting in homologous recombination repair defects. Our findings define a role for DDX5 in facilitating the clearance of RNA transcripts overlapping DSBs to ensure proper DNA repair.

## INTRODUCTION

R-loops are transient, reversible structures consisting of a DNA/RNA hybrid and a displaced single-strand DNA (ssDNA). R-loops participate in a number of physiological processes such as transcription and class switch recombination (1,2). R-loops constitute a major challenge for DNA replication and represent a source of replication stress (3). The persistence of unscheduled R-loops and their collisions with replication fork are known to predispose to double-strand DNA breaks (DSBs) and cause genome instability (4), including chromosomal translocations (5).

There are many processes implicated in the suppression of R-loop formation. Defects in mRNA processing, such as pre-mRNA splicing and mRNA export, accumulate R-loops (6,7). Topoisomerases TOP1 and TOP3B play a key role in maintaining the DNA tension in chromatin during transcription and their deficiency accumulates R-loops (8,9). Many RNA and DNA helicases have been identified to resolve persistent R-loops, including senataxin (SETX) (10,11), aquarius (AQR) (12), BLM (13,14), DDX1 (15,16), DDX5 (17), DDX21 (18), DDX23 (19), DHX9 (20) and PIF1 (21). Another class of enzyme that suppresses R-loops is the RNAse H1 and RNase H2 able to degrade the RNA component in the R-loop (22). Although extensive studies have demonstrated that transcription-associated R-loops can cause DSBs, much remains to be defined about how ongoing transcription and associated R-loop formation neighboring a lesion affect DNA repair (23). DNA damage in *cis* could interfere with gene transcription, splicing and DNA/RNA hybrid formation present in and proximal to the lesion (24).

DSBs are repaired either by non-homologous end joining (NHEJ) or homologous recombination (HDR) (25–28). HDR requires end processing and resection by the MRE11–RAD50–NBS1 (MRN) complex, CtBP-interacting protein (CtIP), exonuclease 1 (EXO1), and DNA Replication Helicase/Nuclease 2 (DNA2) to generate 3′-ssDNA tails coated with the ssDNA-binding protein complex replication protein A (RPA) and subsequently by RAD51 (25,29) and reviewed in (30). R-loop resolution implicates HDR proteins MRN (31), BRCA1 (10) and BRCA2 (32) in the process of preventing accumulation of DSBs. While DNA repair factors are implicated in R-loop biology, the converse is also true (33). Accumulating evidence reveals that RNA and RNA-binding proteins (RBPs) play an important role in the DNA damage response (34). The accumulation of R-loops has been shown to influence resection at DSBs (35), and thus R-loops may influence repair choices. R-loops have also been shown to influence asymmetric resection at DSBs and were proposed to be part of the repair process, as their degradation by RNase H is required for DNA end resection (36). Moreover, local transcription by RNA polymerase II was shown and proposed to be required to maintain the sequence information near DSBs (36). Consistent with this, R-loops forming near a DSB have been shown to lead to asymmetric resection with the side containing the R-loop harboring resection defects (37). In addition, small non-coding RNAs, termed DSB-induced small RNAs or Dicer/Drosha-dependent RNAs, have been identified at sites of DSBs (38–40).

We have shown previously that the DEAD box RNA helicase DDX5 is a key player in resolving persistent unscheduled R-loops (17). Genome-wide DNA/RNA immunoprecipitation (DRIP) sequencing revealed that DDX5-deficient cells have an elevated number of peaks with increased R-loop accumulation at promoters and near the transcription start site causing increased antisense intergenic transcription (41). Furthermore, DDX5-deficient cells have an elevated number of peaks with increased R-loop accumulation at transcription termination site, consistent with its role in transcription termination (41). The expression of DDX5 is elevated in numerous cancer types (42–47) and as such represents a valid cancer therapeutic target. Indeed, a small molecule inhibitor RX-5902 (48) that targets DDX5 interactors is in phase II clinical trial for triple-negative breast cancer. In this paper, we show that DDX5 localizes transiently to R-loops at DSBs to preserve genome integrity. Consequently, DDX5 deficiency leads to R-loop accumulation near DSBs and causes asymmetric end deletions. Therefore, targeting of DDX5 via its ATP-dependent helicase domain or blocking its protein–protein interactions represents therapeutic vulnerabilities to accumulate R-loops and asymmetric deletions, and reduce DNA repair efficiency.

## MATERIALS AND METHODS

### Reagents and antibodies

Mouse anti-DDX5 (A-5, sc-166167) monoclonal and rabbit anti-RAD51 (H-92, sc-8349) and anti-Ku80 (H-300, sc-9034) antibodies were purchased from Santa Cruz Biotechnology (Santa Cruz, CA). Rabbit anti-53BP1 (NB100-304) was from Novus Biologicals (Littleton, CO). Mouse anti-γH2AX (05-636), anti-p68 (DDX5) (clone204, 05-850) monoclonal antibodies, and rabbit anti-BRCA1 antibody (07-434) were obtained from Millipore (Billerica, MA). Anti-cyclin A (BD611268) monoclonal antibody was from BD Biosciences (San Jose, CA). Anti-BrdU antibody (RPN202) was from Cytiva. V5 mouse mAb antibody was from Life Technologies (#R96025). S9.6 antibody was purified, in house, from the hybridoma (ATCC^®^ HB-8730). Propidium iodide and the Alexa Fluor-conjugated goat anti-rabbit antibodies and anti-mouse antibodies were from Invitrogen (Carlsbad, CA). *Escherichia coli* RNase H was purchased from New England Biolabs. Bromo-2′-deoxyuridine (BrdU), 4-hydroxytamoxifen (4-OHT), protein A Sepharose, mouse anti-Flag, and α-tubulin monoclonal antibodies were from Millipore-Sigma (St Louis, MO). For DRIP, Pierce™ Protein A/G UltraLink™ Resin was purchased from Thermo Fisher (#53133). Shield 1 was purchased from Clontech Laboratories (Mountain View, CA). Protease inhibitor cocktail and protein phosphatase inhibitor cocktail were purchased from Roche (Mississauga, ON, Canada).

### Cell culture, treatment and transfection

All mammalian cells were cultured at 37°C with 5% CO_2_. U2OS human osteosarcoma cells (ATCC), U2OS-265 and DRGFP cells were cultured in Dulbecco’s modified Eagle’s medium containing 10% (v/v) fetal bovine serum (FBS). U2OS cells were transfected with plasmid DNAs using Lipofectamine 2000 and siRNA oligonucleotides using Lipofectamine RNAiMAX (Invitrogen) according to the manufacturer’s instructions. HEK293 cells were transfected using the standard calcium phosphate precipitation method. HeLa cells were cultured in Dulbecco’s modified Eagle’s medium containing 10% (v/v) FBS. HeLa cells were transfected with plasmid DNAs using Effectene (Qiagen) and siRNA oligonucleotides using Lipofectamine RNAiMAX (Invitrogen) according to the manufacturer’s instructions.

### siRNAs

All siRNAs were purchased from Dharmacon. siRNA sequences were as follows: siDDX5 #1, 5′-CAA GUA GCT GCU GAA UAU UUU-3′; siDDX5 #2, 5′-CAC AAG AGG UGG AAA CAU AdTdT-3′; si53BP1, 5′-GAA GGA CGG AGU ACU AAU AdTdT-3′; siKu80, Smartpool siGENOME human XRCC5 siRNA (M-010491-00); siBRCA1, Smartpool siGENOME human BRCA1 siRNA (M-003461-02); siBRCA2, Smartpool siGENOME human BRCA2 siRNA (M-003462-01); siRif1, Smartpool siGENOME human RIF1 siRNA (M-027983-01); siMRE11, Smartpool siGENOME human siMRE11 siRNA (M-009271-01); siEXO1, Smartpool siGENOME human siEXO1 siRNA (M-013120-00); siCtIP, Smartpool siGENOME human RBBP8 siRNA (M-011376-00); siBLM, Smartpool siGENOME human BLM siRNA (M-007287-02); and siRNA 5′-CGU ACG CGG AAU ACU UCG AdTdT-3′, targeting the firefly luciferase (GL2), were used as controls (49). siRNA (20 nM) was used. For co-transfection of two or more siRNAs, the total siRNA amount was adjusted to be the same in each sample by adding control siRNA (siLuc, GL2).

### Plasmids

pimEJ5GFP plasmid was purchased from Addgene. pcDNA3.1(+) was obtained from Invitrogen (Carlsbad, CA). pEGFP-C1 and pRetro-tight-Pur plasmids were purchased from Clontech Laboratories (Mountain View, CA). pGEM-T linear plasmid was purchased from Promega Corporation (Madison, MI). The N-terminal Flag-tagged DDX5 plasmid was constructed by inserting a Flag-coding sequence into the pcDNA3.1(+) vector at the *Hind*III and *Bam*HI sites to get pcDNA3.1-Flag and then the PCR-amplified human DDX5 cDNA coding region at *Bam*HI and *Xho*I sites of pcDNA3.1-Flag vector. The Flag-DDX5 codon-silent mutant resistant to all three siDDX5 siRNAs used in this research was constructed in the pcDNA3.1-Flag vector using Gibson Assembly Cloning Kit (New England Biolabs) according to the manufacturer’s instructions. The gBlock DNAs were synthesized by Integrated DNA Technologies. The catalytically inactive DDX5 mutant with replacement of both R403 and R428 with Leu was constructed by two-step PCR using the siRNA-resistant DDX5 construct as a template. GFP-DDX5 plasmid was constructed by inserting human DDX5 cDNA coding region into pEGFP-C1 vector at the *Bgl*II and *Sal*I sites. The Flag-tagged DDX5 truncated and deleted mutants were constructed using standard PCR-based mutagenesis.

The tetracycline-on puro-GFP reporter plasmid (pRetroX-Tight-Pur-GFP) was constructed by inserting a PCR-amplified fragment from pimEJ5GFP reporter plasmid (Addgene, #44026) into pRetroX-Tight-Pur vector. The PCR primers, 5′-GGG GCG GCC GCC ACC ATG GTG AGC AAG GG-3′ (forward) and 5′-GGG GAA TTC TTA CTT GTA CAG CTC GTC CAT GC-3′ (reverse), amplified the reporter fragment containing both I-SceI sites and the puromycin and GFP coding regions. The PCR product was digested using NotI and inserted into pRetroX-Tight-Pur vector at the NotI and EcoRV sites. The puromycin selection marker in the pRetroX-Tight-Pur vector was removed and replaced by the puromycin gene in the insert. ppyCAG_RNAseH1 vector (#111906) and pICE-RNaseHI-WT-NLS-mCherry vector (#60365) were purchased from Addgene.

### Immunofluorescence

Cells growing on glass coverslips were washed with phosphate-buffered saline (PBS) twice and fixed for 10 min with 4% paraformaldehyde (PFA). After three washes, the cells were permeabilized for 5 min with 0.5% Triton X-100 in PBS. Coverslips were incubated overnight in PBS blocking buffer containing 10% FBS and 0.2% Triton X-100, and then incubated with primary antibodies diluted in PBS containing 5% FBS for 30 min. After three washes, the coverslips were incubated with corresponding fluorescent secondary antibodies for another 30 min in PBS buffer containing 5% FBS. After rinsing, the coverslips were mounted with Immuno-Mount (Thermo Scientific) mounting medium containing 1 μg/ml of 4′,6-diamidino-2-phenylindole (DAPI). Images were taken using a Zeiss M1 fluorescence microscope. The percentage of cells containing >10 nuclear fluorescent foci was calculated by manually examining a minimum of 400 cells with >10 images for each slide.

For BrdU/ssDNA detection, U2OS cells were plated on glass coverslip and pre-incubated in the presence of 10 μM BrdU (Sigma) for 36 h followed by a 4 h incubation after ionizing radiation (IR) at 10 Gy. Cells were subjected to *in situ* fractionation on ice for 10 min using sequential extraction with two different buffers. Pre-extraction buffer 1 (10 mM PIPES, pH 7.0, 300 mM sucrose, 100 mM NaCl, 3 mM MgCl_2_ and 0.5% Triton X-100) followed by pre-extraction buffer 2 (10 mM Tris, pH 7.5, 10 mM NaCl, 3 mM MgCl_2_, 1% NP-40 and 0.5% sodium deoxycholate). Cells were washed once time with 1× PBS followed by fixation with 4% PFA (w/v) for 15 min at room temperature. Methanol fixation was then applied for 5 min at −20°C. Cells were washed with PBS and permeabilized in PBS containing 0.5% Triton X-100 for 5 min. Cells were incubated overnight at 4°C with anti-PCNA (1:500, Clone 16D10, Chromotek) and anti-BrdU antibody (1:1000, GE Healthcare, #RPN202) under non-denaturing conditions diluted in PBS containing 3% bovine serum albumin (BSA). In these native conditions, the anti-BrdU antibody has access to its epitope only when DNA is the ssDNA form. Unbound primary antibodies were removed by washing three times for 10 min in PBS at room temperature. Secondary antibodies Alexa Fluor 568 goat anti-rat (Invitrogen, #A-11011) and Alexa Fluor 488 goat anti-mouse (Invitrogen, #A-11001) were diluted 1:400 and 1:1000, respectively, in 1% BSA and incubated for 1 h. Nuclei were stained for 10 min with 1 μg/ml DAPI prior to mounting onto slides with 90% glycerol containing 1 mg/ml *para*-phenylenediamine anti-fade reagent. Images were acquired at 63× magnification on a Leica DMI6000. BrdU foci were counted using CellProfiler software. Results are from three independent experiments and 1000 cells have been counted in total.

### Time-lapse microscopy analysis of laser-induced DNA damage

For recruitment by laser-induced DNA damage, U2OS cells were seeded onto 35-mm fluorodishes (World Precision Instruments, Inc.) and transfected with 2 μg GFP-DDX5 using Lipofectamine 2000 transfection reagent (Thermo Fisher). Cells were untreated or treated with 100 μM of 5,6-dichloro-1-β-d-ribofuranosylbenzimidazole (DRB) for 3 h. For time-lapse microscopy, cells were micro-irradiated using point bleach mode for 200 ms with a 405-nm UV laser (100% output) at the following settings: format 512 × 512 pixels, scan speed 100 Hz, mode bidirectional, zoom 2× and 16-bit image depth. To monitor the recruitment of GFP-DDX5 to laser-induced DNA damage sites, cells were imaged every 20 s for 3.5 min on a Leica TCS SP5 II confocal microscope driven by Leica LAS AF software. The fluorescence intensity of GFP-DDX5 at DNA damage sites relative to an unirradiated area was quantified and plotted over time. Data show the mean relative fluorescence intensity ± standard error (SE) of ∼50 cells per condition from at least three independent experiments. For RPA2, EXO1 and Ku80 recruitment, live-cell imaging and micro-irradiation experiments were carried out with a Leica TCS SP5 II confocal microscope driven by Leica LAS AF software using a 63×/1.4 oil immersion objective. The microscope was equipped with an environmental chamber set to 37°C and 5% CO_2_. Briefly, HeLa cells were seeded and reverse transfected with siCTL or siDDX5 (50 nM) using Lipofectamine RNAiMAX (Invitrogen). Twenty-four hours later, cells were forward transfected with the same siRNA. Cells were seeded onto 35-mm fluorodishes (World Precision Instruments, Inc.) and transfected with 1 μg of GFP-RPA2, GFP-EXO1 or GFP-Ku80 using Effectene transfection reagent (Qiagen), as previously described (50). The next day, cells were micro-irradiated in the nucleus for 200 ms using a 405-nm UV laser at the following settings: format 512 × 512 pixels, scan speed 100 Hz, mode bidirectional and 2× zoom. To monitor the recruitment of indicated protein to laser-induced DNA damage sites, cells were micro-irradiated and imaged every 10 s for 5 min (for RPA2 and EXO1) or 20 s for 10 min (for Ku80). Fluorescence intensity of GFP at DNA damage sites relative to an unirradiated nuclear area was quantified and plotted over time. Kinetic curves were obtained by averaging the relative fluorescence intensity of cells displaying positive recruitment (total *n* > 70 cells) and error bars show the standard error of the mean (SEM). All results are from at least three independent experiments.

### LacI-FokI-mCherry DSB reporter assay

The U2OS LacI-FokI-mCherry DSB reporter cell line was provided by Dr Roger Greenberg (24). Three days after transfection with siRNA or 2 days after transfection with plasmid DNA, the cells were treated with 4-OHT (500 nM) and shield 1 (1/1000 dilution) for 2–4 h and the cells were fixed with 4% PFA and subjected to the immunofluorescence assay as described earlier. In some experiments, the cells were treated with ataxia telangiectasia mutated (ATM), ataxia telangiectasia and Rad3-related (ATR), DNA-dependent protein kinase (DNA-PK), poly(ADP-ribose) polymerase (PARP) or poly(ADP-ribose) glycohydrolase (PARG) inhibitors for 4 h before adding 4-OHT and shield 1. The images were quantified using ImageJ software. The mCherry signal was used to identify the area of damage. The fluorescence intensity of the stained protein at the damage area was determined and the background of the nuclear staining was subtracted for each cell. The results were presented as relative intensity change at the damage site divided by the nuclear staining.

### Clonogenic cell survival assay

For the clonogenic assay of IR-treated cells, 200–800 cells per 10-cm dish were seeded in triplicate 4–16 h after IR treatment. For the clonogenic assay of etoposide-treated cells, 200–800 cells per 10-cm dish were seeded in triplicate first and the cells were treated with etoposide for 3 h. Ten to fourteen days after plating, the cells were fixed with 4% PFA and stained with 0.1% crystal violet (Sigma-Aldrich) and the colonies were counted.

### FACS-based survival analysis

U2OS cells were transfected with pEGFP-C1 plasmid DNA and a single clone of stable cell lines (U2OS-GFP) was selected. The U2OS and U2OS-GFP cells were transfected with control and target siRNAs, respectively. The transfected cells were trypsinized, mixed with an ∼1:1 ratio and co-plated 2 days after transfection. The cells were then treated with DNA damage agents or left untreated. After 7–10 days of recovery, the ratio of GFP+/GFP− cells was assessed using fluorescence-activated cell sorting (FACS) to determine the relative survival of the two mixed cell lines.

### NHEJ assay using reporter system

U2OS cells were transfected with pimEJ5GFP plasmid DNA (51). Both single clones and pools of stable cell lines were generated by growing the cells in medium containing 2 μg/ml of puromycin. The pimEJ5GFP reporter contains two I-SceI endonuclease sites. A puromycin selection marker is located between the two I-SceI sites and a GFP reporter is followed to the 3′-terminal I-SceI site. Induction of DSB at the two sites by I-SceI and rejoining by NHEJ lead to removal of the puromycin selection marker and expression of GFP reporter.

The stable U2OS reporter cells were first transfected with siRNAs. Twenty-four hours after transfection of the siRNAs, the cells were trypsinized and replated in triplicate. Sixteen to twenty hours after replating, the cells were transfected with the plasmid expressing I-SceI restriction enzyme. Two or three days after plasmid transfection, the cells were harvested and genomic DNA was extracted and subjected to PCR and sequencing analysis. Two pairs of primers were designed to amplify the DNA fragments rejoined after cleavage of the two I-SceI sites. The forward and reverse primers used for the first PCR reaction were 5′-GCG CGG CGA GCC GCA GCC ATT GCC-3′ and 5′-TCA GCT CGA TGC GGT TCA CCA GGG-3′. The first PCR product was purified using Qiagen PCR purification kit and used as a template for the second PCR reaction using the following primers: 5′-GCG CAG GGA CTT CCT TTG TCC-3′ and 5′-TCG GCG CGG GTC TTG TAG TTG CC-3′. The PCR condition was 95ºC/5 min, 30 cycles of 95ºC/30 s, 58ºC/30 s and 72 ºC/45 s, and 72ºC/10 min for both first and second PCR reactions. The PCR product was cloned in pGEM-T vector (Promega) and each single clone was selected for sequencing analysis using SnapGene software. The PCR product was also used for quantitative PCR (qPCR) analysis. The primer pairs used for qPCR were 5′-TTC GGC TTC TGG CGT GTG ACC-3′ and 5′-CTT TGC CAA AAT GAT GAG ACA GCA C-3′ for fragment F1, 5′-GTC GCC ACC ATG GTG AGC AAG-3′ and 5′-GCC GGA CAC GCT GAA CTT GTG-3′ for fragment F2, and 5′-TGC AGT GCT TCA GCC GCT ACC-3′ and 5′-GCG GGT CTT GTA GTT GCC GTC-3′ for fragment Fa.

For the inducible NHEJ reporter system, the tetracycline-on puro-GFP reporter plasmid (pRetroX-Tight-Pur-GFP) was transfected in HEK293 cells in which pRetroX-Tet-On advanced (Clontech) vector had been integrated and stable cell lines were generated by selection with puromycin in the presence of doxycycline (Dox). DNA end resection was assessed by qPCR as performed above in the pimEJ5GFP reporter system. The primer pairs used for the first PCR were 5′-CCT CAC TCC TTC TCT AGG CGC CGG-3′ and 5′-TCA GCT CGA TGC GGT TCA CCA GGG-3′, and those for the second PCR were 5′-GAG GCC CTT TCG TCT TCA CTC GAG-3′ and 5′-TCG GCG CGG GTC TTG TAG TTG CC-3′. The PCR product was diluted and used for qPCR analysis. The primer pairs used for amplification of the fragment F1 in qPCR were 5′-GAA CGT ATG TCG AGT TTA CTC CC-3′ and 5′-CAG ATC GCC TGG AGA AGG ATC-3′ and those for fragment F2 were the same primers used for fragment Fa in the pimEJ5GFP system.

### Cross-linking and immunoprecipitation

The cross-linking and immunoprecipitation (CLIP) experiments were performed as described previously (52). Briefly, DRGFP U2OS cells transfected with empty vector (pCAG) or the I-SceI-expressing vector (pCAG-I-SceI) with or without co-transfection with wild-type Flag-DDX5 or Flag-DDX5 catalytic inactive mutant were incubated with 100 μM 4-thiouridine for 8 h prior to cross-linking. The cells were washed with ice-cold PBS and irradiated with 0.15 J/cm^2^ of 365 nm UV light at 4°C. The cells were collected by centrifugation at 514 × *g* for 1 min at 4°C. Cell pellets were resuspended in lysis buffer (50 mM Tris–HCl, pH 7.4, 100 mM NaCl, 1% Igepal CA-630, 0.1% SDS, 0.5% sodium deoxycholate) supplemented with protease inhibitors (Roche) and 0.5 U/ml RNasin (Promega) and sonicated twice with 10 s bursts. Ten microliters of 1:250 dilution of RNase I (Life Technologies) and 2 μl Turbo DNase (Life Technologies) were added to the lysate while shaking at 37°C for 3 min. The lysates were then cleared, 50 μl of the lysates was collected as input and 500 μl was used for immunoprecipitation with 1 μg of anti-DDX5 antibody or mouse IgG and protein A agarose beads (25 μl) for endogenous DDX5 or anti-Flag affinity beads (25 μl) for transfected Flag-tagged DDX5. The beads were washed twice with high-salt buffer (50 mM Tris–HCl, pH 7.4, 1 M NaCl, 1 mM EDTA, 1% Igepal CA-630, 0.1% SDS, 0.5% sodium deoxycholate), twice with the lysis buffer and incubated with Proteinase K buffer (100 mM Tris–HCl, pH 7.4, 50 mM NaCl, 10 mM EDTA) containing 1.2 mg/ml Proteinase K for 20 min at 37°C. RNA was then isolated through TRIzol^®^ reagent and subjected to RT-qPCR to quantify the RNA transcript from the DRGFP reporter gene. The primer (5′-CTG AAC TTG TGG CCG TTT AC-3′) used for the reverse transcription reaction is 10 bp upstream of the I-SceI cleavage site. The qPCR primers (forward primer: 5′-CAG CCC GCC ACC TGC CCC ATC-3′; reverse primer: 5′-CAC CCC GGT GAA CAG CTC CTC-3′) amplify a 150 bp length of fragment at 60 bp upstream of the I-SceI cleavage site. The PCR primers were verified and a standard curve was used to quantify the relative RNA amount. The relative amount RNA bound in the CLIP precipitation was divided by that in the input of the same sample and the data were expressed as percentage of input.

### DRIP-qPCR, ChIP-qPCR and RT-qPCR

For the chromatin immunoprecipitation (ChIP) assays, the cells were cross-linked with 1% formaldehyde at room temperature for 10 min and subsequently quenched by adding glycine to a final concentration of 125 mM. After washing with ice-cold PBS, cells were suspended in lysis/IP buffer (50 mM Tris–HCl, pH 8, 100 mM NaCl, 5 mM EDTA, 0.25% NP-40, 0.25% Triton X-100, 0.25% sodium deoxycholate, 0.05% SDS and protease inhibitors) and sonicated to yield chromatin fragments of 300–1000 bp. After pelleting debris, the supernatants were precleared with protein G beads (Sigma) for 1 h. The precleared chromatin extract was incubated overnight with 5 μg of the indicated antibodies. Protein G was added for the final 2 h of incubation. The beads were washed with the following buffers: low-salt wash buffer (0.1% SDS, 1% Triton X-100, 2 mM EDTA, 20 mM Tris–HCl, pH 8.0, 150 mM NaCl), medium-salt wash buffer (0.1% SDS, 1% Triton X-100, 2 mM EDTA, 20 mM Tris–HCl, pH 8.0, 300 mM NaCl), high-salt wash buffer (0.1% SDS, 1% Triton X-100, 2 mM EDTA, 20 mM Tris–HCl, pH 8.0, 500 mM NaCl), LiCl wash buffer (250 mM LiCl, 1% NP-40, 1% sodium deoxycholate, 1 mM EDTA, 10 mM Tris–HCl, pH 8.0) and twice with TE buffer (10 mM Tris–HCl, pH 8.0, 1 mM EDTA). DNA was eluted from the beads with elution buffer (0.1 M NaHCO_3_, 1% SDS) at 65°C. After the cross-linking was reversed at 65°C overnight in the presence of 200 mM NaCl, the eluted chromatin as well as the input was treated with Proteinase K for 1 h at 45°C. After phenol–chloroform extraction, the DNA was precipitated with ethanol in the presence of 20 μg glycogen. The enriched chromatin was analyzed by qPCR using the following primers.

Primers used in HEK293 tet-puro-I-SceI-GFP reporter region were as follows: position 1 (P1) primer pair, 5′-GAA CGT ATG TCG AGT TTA CTC CC-3′ and 5′-GAT CCT TCT CCA GGC GAT CTG-3′; position 2 (P2) primer pair, 5′-TTC GGC TTC TGG CGT GTG ACC-3′ and 5′-CTT TGC CAA AAT GAT GAG ACA GCA C-3′; and *MDM2* promoter primer pair, 5′-GGT TGA CTC AGC TTT TCC TCT TG-3′ and 5′-GGA AAA TGC ATG GTT TAA ATA GCC-3′. The primers for 5′ *LMNA* homology arm were as follows: 5′-GCG TCG GTG ACT CAG TGT T-3′ and 5′-GGT CGA AGG ACA GAG ACT GC-3′; 3′ *LMNA* homology arm: 5′-ACC TGC AGG AGC TCA ATG AT-3′ and 5′-AAC TCC TCA CGC ACT TTG CT-3′. For DRIP-qPCR assays, they were performed as previously using the *EGR1* locus as a positive control (17). The *LMNA* primers were the same as above for DRIP-qPCR.

For RT-qPCR, total RNA was isolated from the DRGFP reporter cells (53) using GenElute Mammalian Total RNA Miniprep Kit (Sigma-Aldrich) and cDNA synthetized using M-MLV reverse transcription kit (Promega) and random primers according to the manufacturers’ instructions. GAPDH housekeeping gene expression was used to normalize the expression of the reporter gene.

### CRISPR-LMNA HDR assays

For the CRISPR-LMNA HDR assay (54) without RNase H treatment, U2OS cells were seeded and reverse transfected with siDDX5 or siCTL (50 nM) in six-well plates at 2 × 10^5^ cells per well using Lipofectamine RNAiMAX (Invitrogen). Twenty-four hours later, cells were transfected with the same siRNA. Twenty-four hours post-transfection, 2 × 10^6^ cells were pelleted for each condition and resuspended in 100 μl complete nucleofector solution (SE Cell Line 4D-Nucleofector™ X Kit, Lonza) to which 1 μg of pCR2.1-mClover2LMNAdonor, 1 μg pX330-LMNAgRNA and 0.1 μg of 2XNLS-piRFP were added. Once transferred to a 100 μl Lonza certified cuvette, cells were transfected using the 4D-Nucleofector X-unit, program CM-104 and plated onto glass coverslips. Twenty-four hours post-transfection, cells were fixed with 4% PFA for 10 min, followed by 5 min in methanol at −20°C. This was succeeded by permeabilization in 0.2% Triton X-100 for 5 min and a quenching step using 0.1% sodium borohydride for 5 min. After blocking for 1 h in a solution containing 10% goat serum and 1% BSA, cells were incubated for 1 h with primary anti-cyclin A antibody (1:400, BD Biosciences, #611268) diluted in 1% BSA. For the CRISPR-LMNA HDR assay in the presence of RNAse H1, U2OS or HEK293 cells were seeded and reverse transfected with siDDX5 or siCTL (10 nM) in 10-cm dish using Lipofectamine RNAiMAX (Invitrogen). Twelve hours later, cells were transfected with either pcDNA3 or ppyCAG_RNAseH1 vector (Addgene, #111906) with Lipofectamine 2000 (Invitrogen). Medium was changed 4 h later. Twenty-four hours post-transfection, 4 × 10^6^ U2OS cells were subjected to nucleofection as described earlier using 1.5 μg of pCR2.1-mClover2LMNAdonor, 1.5 μg pX330-LMNAgRNA and 0.15 μg of 2XNLS-piRFP. HEK293T cells were transfected with 14 μg of pCR2.1-mClover2LMNAdonor, 14 μg of pX330-LMNAgRNA and 1.4 μg of 2XNLS-piRFP using the standard calcium phosphate precipitation method. Twelve hours after transfection, cells were seeded on glass coverslips. For both U2OS and HEK293, cells were fixed with 4% PFA for 15 min 48 h after CRISPR-LMNA assay vector transfection. This was succeeded by permeabilization in 0.25% Triton X-100 for 10 min. After blocking for 1 h in PBS containing 5% BSA, cells were incubated for 2 h with primary anti-V5 antibody (1:400, Life Technologies, #R96025) diluted in PBS containing 1% BSA. In all IF, Alexa Fluor 568 goat anti-mouse antibody (Invitrogen, #A-11004) was then diluted 1:1000 in 1% BSA and applied for 1 h. Nuclei were stained for 10 min with 1 μg/ml DAPI prior to mounting onto slides with 90% glycerol containing 1 mg/ml *para*-phenylenediamine as anti-fade reagent. Images were acquired on a Leica CTR 6000 microscope using a 63× oil immersion objective and analyzed for mClover and cyclin A or V5 tag expression on a Leica CTR 6000 inverted microscope using a 63×/1.40 oil immersion objective. mClover-LMNA-positive cells were quantified among iRFP-positive cells or iRFP and V5-positive cells. As RNH1 expression is decreasing the percentage of mClover-positive cells in control condition, percentage of Clover-LMNA-positive cells was normalized to siCTL condition as 100% for both pcDNA and RNAse H conditions. Results are from three independent experiments and at least 150 iRFP or iRFP and V5 positive cells were counted in each replicate.

### Statistical analysis

All differences between treatment groups were determined via two-tailed Student’s *t*-test. Significance was indicated as *P < 0.05, **P < 0.01 and ***P < 0.001.

## RESULTS

### DDX5 is excluded from DNA damage sites in transcription-dependent manner

**Figure 1.**
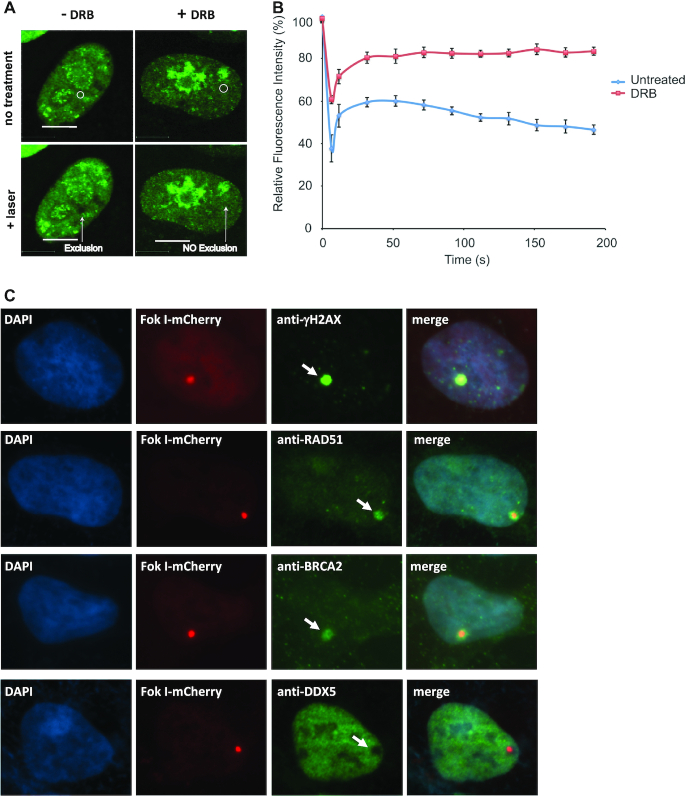
DDX5 is excluded from DNA damage sites in a transcription-dependent manner. U2OS cells transfected with GFP-DDX5 were untreated or treated with 100 μM of DRB for 3 h. The cells were then micro-irradiated and imaged every 20 s for 3.5 min as described in the ‘Materials and Methods’ section. (**A**) A typical image was shown for each sample. (**B**) The fluorescence intensity of GFP-DDX5 at DNA damage sites relative to an unirradiated area was quantified and plotted over time. Data show the mean relative fluorescence intensity ± SE of ∼50 cells per condition from at least three independent experiments. (**C**) The cells transfected with the mCherry-LacI-FokI reporter system were treated with OHT and shield 1 for 4 h and the cells were fixed and stained with specific antibodies as described in the ‘Materials and Methods’ section.

Our previous studies demonstrated that DDX5 resolves R-loop structures and suppresses cellular R-loop accumulation (17,41). In particular, using the DART (DNA damage at RNA transcription) assay, we found that DDX5 deficiency led to a significant increase of R-loop at DNA damage sites (17). To investigate the potential function of DDX5 in the DNA damage repair pathway, we analyzed its localization upon laser-induced DNA damage. Interestingly, we identified that GFP-DDX5 was excluded at sites of laser-induced damage (Figure 1A and B). This exclusion was sensitive to transcriptional inhibition as treatment with the RNA polymerase II inhibitor DRB impaired its exclusion (Figure 1A and B). We next co-transfected U2OS cells with expression vectors encoding GFP-DDX5 and RNAse H1 and examined the exclusion of GFP-DDX5 from sites of laser-induced DNA damage by live-cell microscopy. The presence of RNAse H1 prevented the exclusion of the GFP-DDX5 from laser-induced DNA damage sites (Supplementary Figure S1A). Taken together, our findings suggest that the dissociation of DDX5 from DNA damage sites requires the presence of RNA in the form of DNA/RNA hybrids.

### DDX5 recruitment at DSBs is regulated by ATM

**Figure 2.**
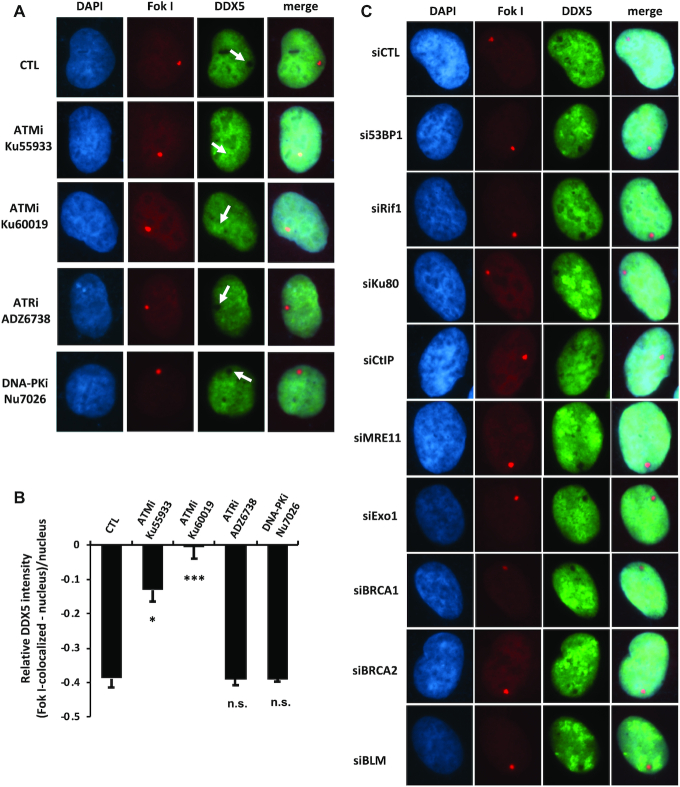
ATM activity regulates DDX5 at DSBs. Cells harboring the mCherry-LacI-FokI reporter system were treated with the inhibitors of the three PIKK family of kinases (ATM, ATR or DNA-PK) (**A**, **B**) or transfected with indicated siRNAs (**C**). DDX5 exclusion from the FokI-induced DSBs was analyzed as described in the ‘Materials and Methods’ section. A typical image is shown for each sample. The fluorescence intensity of DDX5 at DNA damage sites relative to an unirradiated area was quantified. Data show the mean relative fluorescence intensity ± SE of ∼120 cells per condition from three independent experiments. Statistical significance was assessed using Student’s *t*-test: *P < 0.05; ***P < 0.001; n.s., not significant.

We next used the mCherry-LacI-FokI reporter system to define which known DSB factors are required for DDX5 exclusion. The genome of the reporter cells contains several hundred repeats of the Lac operator (LacO) and inducible expression of an mCherry-tagged Lac repressor (LacI)-FokI endonuclease fused to a destabilization domain and a modified estradiol receptor (DD-ER-mCherry-LacI-FokI) results in binding of LacI to the LacO, where the nonspecific FokI endonuclease creates DSBs (24). Using this reporter, we observed an increase in γH2AX and the recruitment of RAD51 and BRCA2, while DDX5 was excluded at DSBs (Figure 1C). We next examined whether DNA damage proteins could regulate DDX5 exclusion. We initially tested the requirement for the three phosphatidylinositol 3-kinase-related kinase (PIKK) family members, ATM, ATR and DNA-PK, using specific inhibitors. DDX5 exclusion from the DSBs required ATM, but not ATR nor DNA-PK, as exclusion was prevented with ATM inhibitors Ku55933 and Ku60019, but not ATR inhibitor ADZ6738 nor with DNA-PK inhibitor Nu7026 (Figure 2A, quantified in Figure 2B). The depletion of DNA damage response proteins including 53BP1, RIF1, Ku80, CtIP, MRE11, EXO1, BRCA1, BRCA2 and BLM did not have significant effect on the ability of DDX5 to be excluded from DSBs (Figure 2C).

We next examined whether DDX5 was an ATM substrate. DDX5 harbors two SQ and two TQ sites (KSQQ, GYSQ, KTQN, STQQ), but none are reported to be phosphorylated on PhosphoSitePlus^®^ nor were they identified in ATM/ATR substrate proteomic screens using many phosphorylated SQ/TQ antibodies (55). Furthermore, our Flag-DDX5 immunoprecipitations were not recognized with the commercial pSQ/TQ antibodies by immunoblotting (data not shown). Next, we proceeded to test whether an ATM inhibitor influenced the kinetics of DDX5 exclusion from DSBs. ATM inhibition did not influence the initial GFP-DDX5 exclusion, but it increased the recovery of GFP-DDX5 at laser-induced DNA damage sites (Supplementary Figure S1B). Although GFP-DDX5 recovered slower immediately after the breaks in the ATMi-treated cells, it kept on recovering over time, while in the control condition the curve keeps going down showing an active exclusion process over time (Supplementary Figure S1B). For example, 4 min after the laser pulse, the DDX5 signal in the ATM inhibitor-treated cells recovered to 55% of the ground signal compared to ∼35% in the control cells (Supplementary Figure S1B). These findings suggest that ATM activation is required for DDX5 exclusion and ATM regulates the return of DDX5 to DNA damage sites.

### DDX5 RGG/RG motif is required for exclusion from DSBs

**Figure 3.**
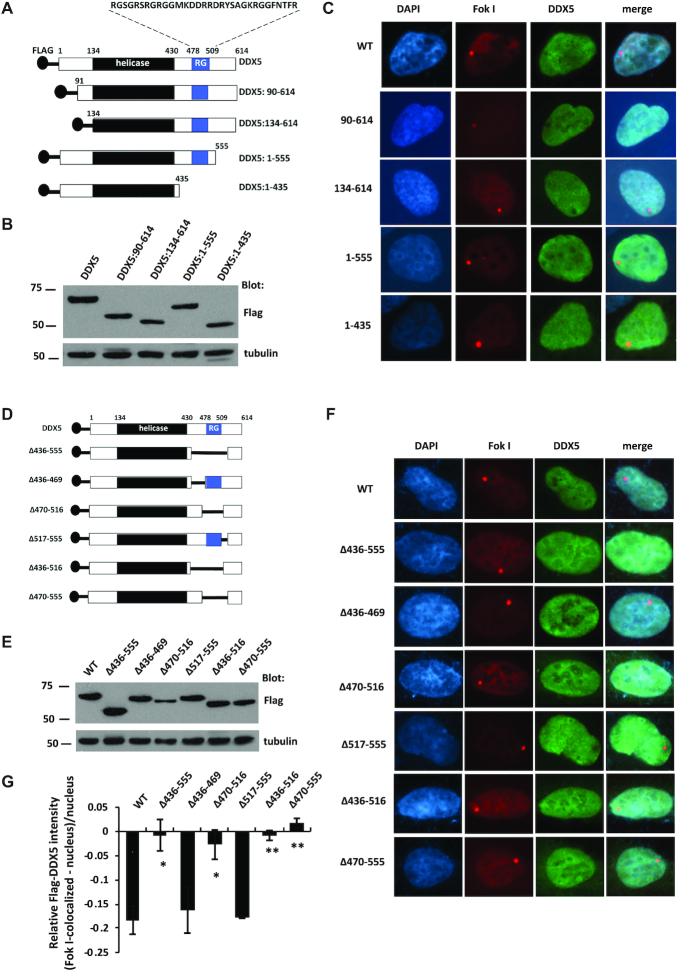
The region containing the RGG/RG motif is required for DDX5 exclusion from DSBs. (**A**, **D**) A scheme of DDX5 functional domains and mutant constructs. (**B**, **E**) Cells harboring the mCherry-LacI-FokI reporter system were transfected with Flag-tagged DDX5 mutants. The whole cell lysates were subjected to western blotting with the indicated antibodies. (**C**, **F**, **G**) DDX5 exclusion from the FokI-induced DSBs was analyzed as described in the ‘Materials and Methods’ section. A typical image is shown for each sample. The fluorescence intensity of DDX5 at DNA damage sites relative to an unirradiated area was quantified. Data show the mean relative fluorescence intensity ± SE of ∼120 cells per condition from three independent experiments. Statistical significance was assessed using Student’s *t*-test: *P < 0.05 and **P < 0.01.

We then performed deletion analysis to identify the DDX5 regions required for its exclusion from DSBs. DDX5 is composed of an ATP-dependent helicase domain (134–430) and a C-terminal RGG/RG motif (478–509 amino acids; Figure 3A). DDX5 was excluded from DSBs and did not require its 133 amino acids N-terminal of the helicase domain nor its most C-terminal 59 amino acids (see proteins DDX5:90–614, DDX5:134–614 and DDX5:1–555; Figure 3B and C). However, deletion of the C-terminal 179 amino acids removing the RGG/RG motif prevented its exclusion (Figure 3C; DDX5:1–435). We next performed shorter internal deletions and found that the deletion of DDX5 amino acids 436–516, 436–555, 470–516 or 470–555 prevented exclusion (Figure 3D–G). Deletion of the amino acids flanking the RGG/RG motif including amino acids 436–469 or 517–555 maintained exclusion (Figure 3F and quantified in Figure 3G).

### DDX5 binds transcribed RNA near DSBs

**Figure 4.**
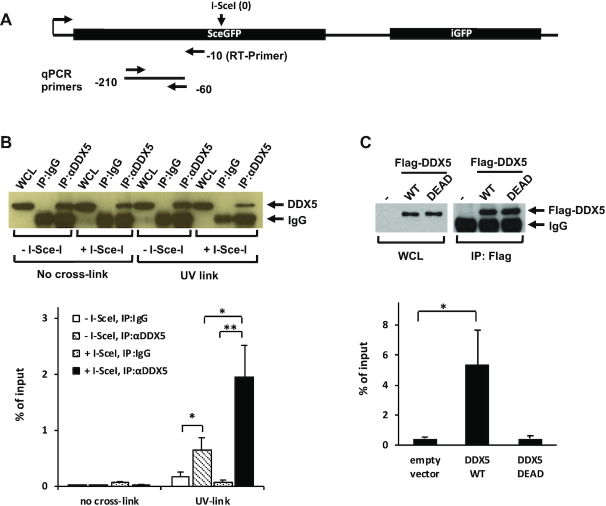
I-SceI-mediated DSBs increase DDX5 binding to the RNA of the *DRGFP* reporter gene. (**A**) Illustration of primers used for reverse transcription (RT) and qPCR analysis. The RT primer is 10 bp upstream of the I-SceI cleavage site (−10). The qPCR primers amplify a 150 bp length of fragment at 60 bp upstream of the I-SceI cleavage site (−60). (**B**) DRGFP cells were transfected with empty vector (−I-SceI) and the I-SceI-expressing vector (+I-SceI), respectively, and then subjected to CLIP analysis as described in the ‘Materials and Methods’ section. The results were expressed as percentage of input. The graph shows the average and SEM from four independent experiments performed in triplicates. A typical western blot analysis shows the DDX5 immunoprecipitated with the antibodies. (**C**) DRGFP cells were co-transfected with the I-SceI-expressing vector, siDDX5 siRNA and siRNA-resistant Flag-tagged wild-type DDX5 or its catalytic inactive mutant (DDX5 DEAD). Cells were subjected to CLIP analysis. The graph shows the average and SEM from three independent experiments performed in triplicates. A typical western blot analysis shows the Flag-DDX5 immunoprecipitated with the anti-Flag antibody. Statistical significance was assessed using Student’s *t*-test: *P < 0.05 and **P < 0.01.

The DDX5 exclusion in a transcription-dependent manner near DSBs implied that DDX5 may bind the transcribed RNAs and facilitate their clearing to prevent them from hindering repair at the break. Thus, we performed CLIP followed by RT-qPCR analysis to detect DDX5-associated RNAs. The DRGFP is a reporter gene with ongoing transcription from the CMV promoter of cDNAs encoding a partial GFP protein and this overlaps an I-SceI site that creates a DSB with I-SceI (Figure 4A) (53). CLIP data revealed that DDX5 associated with ∼0.64% of GFP transcript in the control cells (−I-SceI, IP: anti-DDX5, UV link), while the control IgG bound <0.16%, suggesting that DDX5 associated with GFP RNA in cells (Figure 4B). Importantly, expression of I-SceI to trigger the DSB led to ∼3-fold increase of the GFP RNA bound to DDX5 (from 0.64% to 1.93% of input; Figure 4B). Without UV cross-linking, the GFP RNA was not bound to DDX5 (Figure 4B, no cross-link). RT-qPCR showed a slight decrease of the GFP reporter expression in the I-SceI-transfected cells compared to control plasmid-transfected cells (Supplementary Figure S2), suggesting that the increase of GFP RNA pulled down by DDX5 in the I-SceI-transfected cells was not caused by its increased expression. We next transfected Flag-DDX5 (DDX5 WT) and its catalytically inactive mutant (DDX5 DEAD) in cells to see whether the helicase domain was required for association. Indeed, we observed that the GFP RNA was co-immunoprecipitated with wild-type Flag-DDX5, but not helicase-inactive DDX5 (Figure 4C). These findings suggest that DDX5 associates with RNAs neighboring DSBs and its helicase activity is required for this association.

### DDX5 deficiency causes polarized end deletions at DSBs

**Figure 5.**
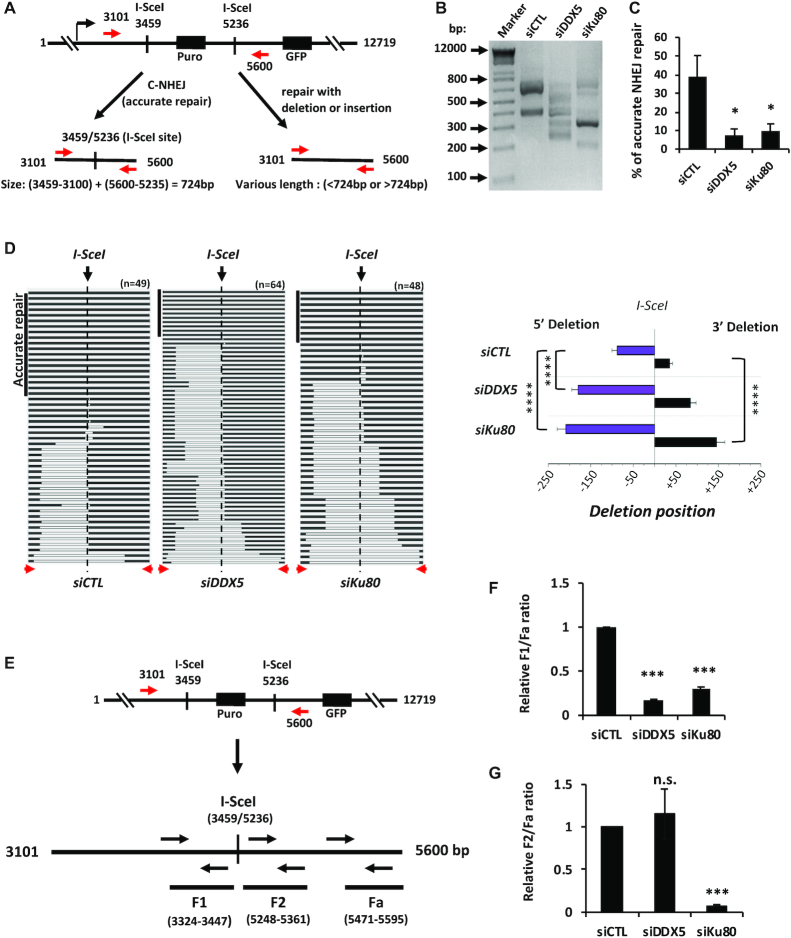
Deficiency of DDX5 causes extended deletions at DSB sites. (**A**) Illustration of the EJ5-GFP reporter system for NHEJ repair analysis and demonstration of the PCR reaction for amplification of the reporter DNA rejoined after cleavage of the two I-SceI sites. The PCR primers amplify a DNA fragment with a size of 724 bp if the two ends are accurately repaired. Under the PCR reaction condition, the DNA (2500 bp) without cleavage cannot be amplified. The U2OS-EJ5-GFP reporter cells were transfected with indicated siLuc control siRNA (siCTL), siDDX5 #1 (siDDX5) and siKu80, respectively. Forty to forty-four hours after the siRNA transfection, the cells were then transfected with I-SceI-expressing vector (pCAG-I-SceI). Forty-eight hours after the plasmid transfection, the cells were harvested and genomic DNA extracted for PCR and sequencing analysis. (**B**) A representative agarose gel analysis of the PCR products. (**C**) The PCR products were subcloned in pGEM-T vector and individual clones were subjected to sequencing analysis. The graph shows the percentage of clones with accurate repair performed from four independent experiments. (**D**) Graphical representation of the extended deletions flanking the I-SceI cut site in siCTL, siDDX5 and siKu80 cells. On the left side is a schematic representation of the sequence alignment obtained with the cloned PCR products from panel (**B**). The horizontal black boxes indicate matched reads, while the white boxes indicate deletions. The red arrowheads indicated the position of the PCR primers. The top left vertical black box indicated reads with accurate repair. On the right is the quantification of deletions identified by DNA sequencing individual clones (*n* represents the number of individual clones sequenced: *n* = 49 for siCTL, *n* = 64 siDDX5 and *n* = 48 for siKu80). ****P < 0.0001. (**E–G**) The PCR products amplified with the primers as in (**A**) were subjected to qPCR analysis targeting different regions surrounding the I-SceI sites. The ratio of each fragments to the fragment Fa was normalized to the one in the siCTL sample. SEM from two independent experiments performed in triplicates. Statistical significance was assessed using Student’s *t*-test: *P < 0.05; **P < 0.01; ***P < 0.001; n.s., not significant.

We then monitored the DNA repair of the EJ5 reporter system, where ongoing transcription overlaps I-SceI sites (51,56). PCR flanking the I-SceI site amplifies a DNA fragment of 724 bp if the DSB is accurately repaired after cleavage of the two I-SceI sites. U2OS cells transfected with siDDX5 or siKu80 exhibited significant increases of PCR products less than the accurate repair product of 724 bp (Figure 5B), indicating deletions were occurring flanking the I-SceI sites. Sequencing of these DNA fragments showed that accurate repair dramatically decreased in the siDDX5 cells (7.4%) and siKu80 cells (9.7%) compared to siCTL cells (38.9%, Figure 5C). Interestingly, sequence analysis revealed that frequent long-range deletions occurred at the 5′-terminal I-SceI site (the site at base pair position 3459; Figure 5A), but less at the 3′-terminal I-SceI site (the site at base pair position 5236; Figure 5A) in siCTL and siDDX5 cells (Figure 5D). For example, in the siCTL, >35% clones displayed deletion at the 5-terminal I-SceI site only, while 5% clones had deletion at both sites, and only one clone showed a long deletion at 3′-terminal I-SceI site (Figure 5D). In contrast, siDDX5 cells dramatically increased the number of clones with deletions at the 5′-terminal site (Figure 5D). siKu80 caused increase of deletions at both 5′-terminal and 3′-terminal I-SceI sites, as expected for Ku80 involved in NHEJ (57).

We then performed qPCR experiments to estimate the deletion of I-SceI-induced DSBs using three pairs of primers (F1, F2, Fa) targeting different regions of the reporter DNA (Figure 5E). The F1 and F2 DNA fragments lie close to the 5′-terminal and 3′-terminal I-SceI cleavage sites, respectively (∼10 bp apart from the cleavage site). The F1 fragment cannot be amplified when DNA end deletion occurs at 5′-terminal I-SceI cleavage site and thus the ratio of F1/Fa decreases. Similarly, F2 fragment cannot be amplified when DNA end deletion occurs at the 3′-terminal I-SceI cleavage site and the ratio of F2/Fa decreases. As shown in Figure 5F, compared to the control, deficiency of DDX5 and Ku80 led to 5-fold and 4-fold decrease of F1/Fa ratio, respectively, suggesting increased deletion at the 5′-terminal I-SceI cleavage site. Deficiency of Ku80 also caused significant decrease of F2/Fa ratio, not observed in siDDX5 cells (Figure 5G).

### DDX5 deficiency causes polarized end deletions at DSBs with ongoing transcription

**Figure 6.**
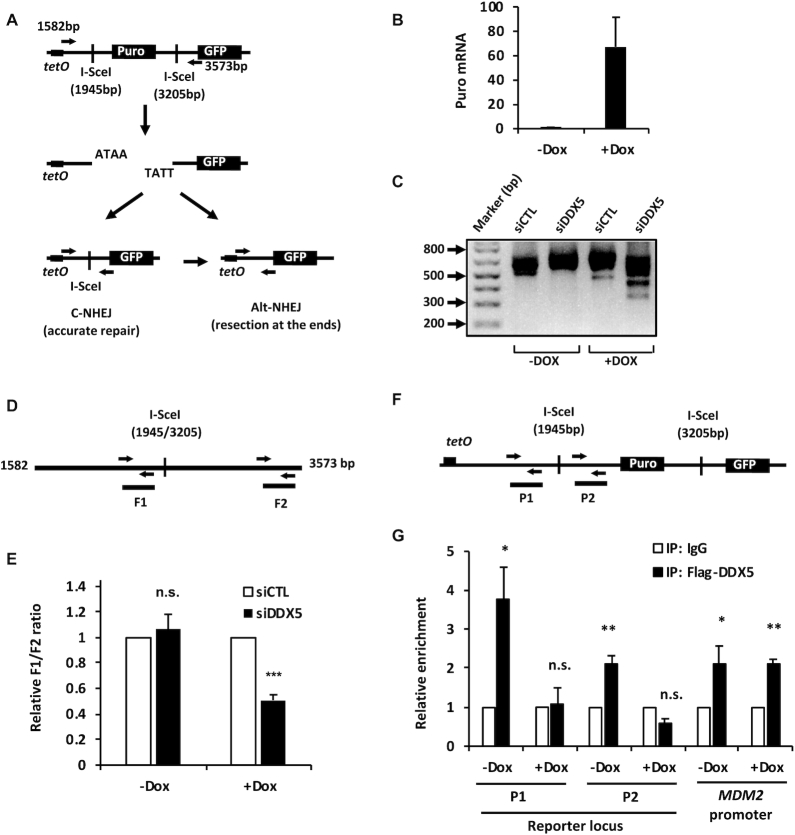
Increased DNA end deletions in DDX5-deficient cells are associated with local gene transcription. (**A**) Illustration of a tetracycline-induced (tetO) reporter system for NHEJ repair analysis in HEK293. The reporter construct is similar to the one described in Figure 4A except that the reporter expression is controlled by a tet-on promoter. (**B**) RT-qPCR analysis of the expression of puromycin in the absence (−Dox) or presence (+Dox) of 1 μg/ml Dox. (**C**) The HEK293-tetO-puro-GFP reporter cells were transfected with indicated siLuc control siRNA (siCTL) and siDDX5 #3 (siDDX5), respectively, in the absence (−Dox) or presence (+Dox) of 1 μg/ml Dox. Forty to forty-four hours after the siRNA transfection, the cells were then transfected with I-SceI-expressing vector (pCAG-I-SceI). Seventy hours after the plasmid transfection, the cells were harvested and the genomic DNA was extracted for PCR analysis using the primers shown in (**A**). The PCR primers amplify a DNA fragment with a size of 733 bp if the two ends are accurately repaired. Under the PCR reaction condition, the DNA (3573–1581 equals 1992 bp) without cleavage cannot be amplified. A representative agarose gel was shown for the analysis of the PCR products. (**D**) The PCR products amplified with the primers as in (**A**) were subjected to qPCR analysis targeting different regions surrounding the I-SceI sites. The ratio of F1/F2 that was normalized to the one in the siCTL sample. (**E**) The graph shows the average and SEM from three independent experiments performed in triplicates. (**F**, **G**) The HEK293-tetO-puro-GFP reporter cells were co-transfected with Flag-DDX5 and I-SceI-expressing plasmids in the absence (−Dox) or presence (+Dox) of 1 μg/ml Dox. ChIP-qPCR was performed to determine DDX5 occupancy near the I-SceI-cleaved DNA breaks (P1 and P2). *MDM2* promoter region was used as a positive control. The results were normalized to IgG control at each condition. The graph shows the average and SEM from four independent experiments. Statistical significance was assessed using Student’s *t*-test: *P < 0.05; *P < 0.01; ***P < 0.001; n.s., no significant.

We noted that the transcript from puromycin-resistant gene (Puro^R^) overlaps the first I-SceI site and there is no transcriptional unit at the second I-SceI site. To test whether transcription was responsible for polarized deletions, we generated a tetracycline-inducible (tet-on) reporter termed tetO-puro-GFP where transcription of Puro^R^ is controlled (Figure 6A). Stable clones were selected using puromycin in the presence of Dox. Expression of Puro^R^ could be induced by >60-fold with 1 μg/ml of Dox (Figure 6B). In the presence of Dox, siDDX5 caused a significant increase of smaller DNA fragments amplified that were not observed in the absence of Dox (Figure 6C). The PCR fragments were subjected to qPCR analysis using the primers that amplify the DNA fragment 16 bp before the 5′-terminal I-SceI site (F1, 1805–1929) and 240 bp after the 3′-terminal I-SceI site (F2, 3444–3568), respectively (Figure 6D). DDX5 deficiency led to significant decrease of the ratio of F1/F2 amplification in the presence of Dox, but had no effect in the absence of Dox (Figure 6E). These findings suggest that the function of DDX5 may be to clear RNA, generated by local transcription in *cis*, to allow proper DNA repair at nearby DSBs.

We then performed ChIP-qPCR analysis to determine whether DDX5 was localized near the DSBs. Cells containing the tetO-puro-GFP reporter were co-transfected with I-SceI and Flag-DDX5 plasmids. The transfected cells were treated with Dox for 24 h or left untreated followed by ChIP-qPCR analysis (Figure 6F and G). In the absence of Dox, DDX5 was enriched ∼4-fold and ∼2-fold over IgG at the P1 and P2 regions and this chromatin association was lost with the addition of Dox to induce transcription (Figure 6G, P1, P2). As a control, we included the p53 target gene *MDM2* in the ChIP-qPCR analysis and Dox treatment did not affect Flag-DDX5 binding to the *MDM2* promoter region (Figure 6G). Taken together, DDX5 associates with genomic DNA near the I-SceI locus and it is released in response to DSBs in a transcription-dependent manner.

### DDX5 deficiency causes IR sensitivity and defects in HDR

**Figure 7.**
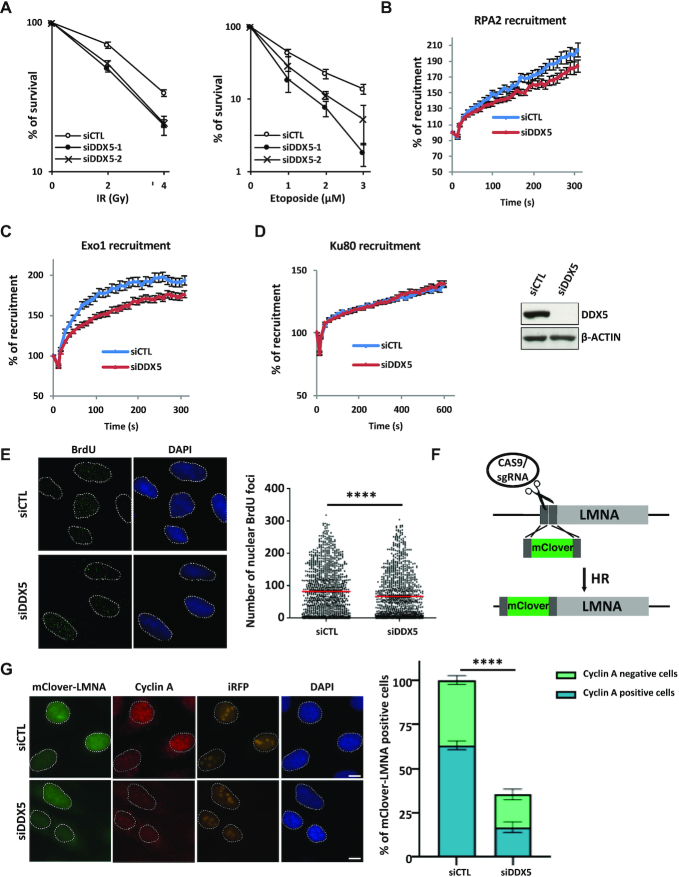
DDX5 deficiency affects HDR. (**A**) U2OS cells were transfected with the siCTL, siDDX5-1 or siDDX5-2. Three days after transfection, the cells were treated with different dosage of IR or etoposide (3 h) as indicated or left untreated. Colony survival analysis was performed as described in the ‘Materials and Methods’ section. The graph shows the average and SEM from three independent experiments performed in triplicates. (**B–D**) Seventy-two hours after siRNA transfection, recruitment of GFP-RPA2 (**B**), GFP-EXO1 (**C**) or GFP-Ku80 (**D**) to laser-induced DNA damage was analyzed for indicated times after damage. The graph shows the mean ± SEM from at least three independent experiments totalizing at least 75 cells. (**E**) Seventy-two hours after siRNA transfection, irradiated U2OS cells (10 Gy, 4 h release) were subjected to BrdU staining. Graph shows mean ± SEM from three replicates. (**F**) The LMNA assay: a specific Lamina sgRNA induced targeted cutting of the Lamin A gene by the Cas9, creating a DSB. When the DSB is repaired by HDR, the donor DNA, which includes mClover sequence flanked by two homology regions corresponding to each side of the site of cutting, is inserted in the Lamin A gene, leading to an mClover LMNA fluorescent protein. (**G**) Forty-eight hours after siRNA transfection, U2OS cells were transfected with the CRISPR–Cas LMNA HDR system and iRFP plasmids. Twenty-four hours later, cells were fixed and subjected to immunofluorescence against cyclin A. Clover-positive cells among iRFP-positive cells were quantified and cyclin A status (positive or negative) was assigned to each cell. The experiment was performed three times, with at least 250 cells counted for each replicate. ****P < 0.0001.

We performed clonogenic survival analysis to measure the cell sensitivity to IR- and etoposide-induced DNA damage. siDDX5 U2OS had reduced cell survival compared with siCTL cells (Figure 7A). We also performed a FACS-based survival analysis of co-cultured cells (outlined in Supplementary Figure S3A). This analysis enables direct comparison of the cell survival of the control and DDX5-deficient cells under the same cell culture conditions. GFP-negative and GFP-positive U2OS cells were transfected with control and DDX5 siRNAs, respectively. The cells were mixed with ∼50% of each and treated with etoposide. Compared to non-treated cells, a decrease in the percentage of GFP-positive cells after treatment indicated that siDDX5-transfected cells (GFP-positive) were more sensitive than the siCTL cells (GFP-negative; Supplementary Figure S3B, right panels). As control, both GFP-negative and GFP-positive cells were transfected with siCTL and treatment with etoposide did not affect the ratio of GFP-positive cells (Supplementary Figure S3B, left panels). Depletion of DDX5 caused significant increase of cell sensitivity to etoposide (decrease of GFP-positive cells), either at low dose (0.4 μM) with long-time incubation (24 h) or at high dose (2 μM) and short exposure (2 h; Supplementary Figure S3C), similar to siBRCA1-transfected cells. In contrast, si53BP1 cells were less sensitive to etoposide (Supplementary Figure S3C, slight increase of GFP-positive cells in the etoposide-treated samples compared to non-treated samples). The double depletion of DDX5 and BRCA1 did not cause further sensitivity (Supplementary Figure S3C).

We next used laser micro-irradiation to study the recruitment of the ssDNA-binding protein RPA2 at DSBs. Depletion of DDX5 led to a delay of RPA2 recruitment and reduction of its persistent retention (Figure 7B). Similarly, DDX5 depletion also caused reduction of the EXO1 recruitment (Figure 7C), but had no effect on the recruitment of Ku80 (Figure 7D). The reduced EXO1 recruitment to the DNA damage sites indicated a reduction in DNA end resection. Next, we asked whether there were less ssDNAs generated (resection) at the DSBs in DDX5-deficient cells. U2OS cells were grown in the presence of BrdU for 24 h, treated with IR and stained using an anti-BrdU antibody without denaturation. This method reveals DNA end resection because the anti-BrdU antibody only labels the ssDNA (50). Indeed, siDDX5 cells had less BrdU foci formation, suggesting reduced resection (Figure 7E).

We next carried out an HDR assay to examine whether reduced resection correlated with DNA damage repair defects. We carried out the HDR assay using the CRISPR–Cas LMNA system (54). In this assay, cells are transfected with plasmids coding for spCas9 and an sgRNA targeting the *Lamin A* or *LMNA* gene that encodes the LMNA protein of the nuclear envelope, while spCas9 will induce a DSB in the *LMNA* gene. In HDR-proficient conditions, DSB repair will lead to the insertion of an mClover coding sequence at the beginning of the *LMNA* gene, leading to fluorescent mClover-LMNA (Figure 7F). Furthermore, to avoid bias induced by nucleofection variability, along with the mClover-LMNA system, cells were also co-transfected with iRFP cDNA to label nucleofected cells. Twenty-four hours later, we assessed the proportion of Clover-positive cells over iRFP-positive cells, which is representative of HDR efficiency. A significant reduction (∼70%) of HDR repair efficiency was observed in siDDX5 cells (Figure 7G). Immunostaining with anti-cyclin A antibodies that stain S/G_2_ phases of the cell cycle did not show a strong difference in cell cycle-dependent appearance of Clover-positive cells. These findings show that siDDX5 U2OS cells exhibit HDR defects.

### Unresolved R-loops impair HDR at the *LMNA* locus

The aforementioned role of DDX5 in HDR led us to hypothesize a mechanism by which DDX5 RNA helicase activity is necessary to remove R-loops near the *LMNA* locus to facilitate HDR.

**Figure 8.**
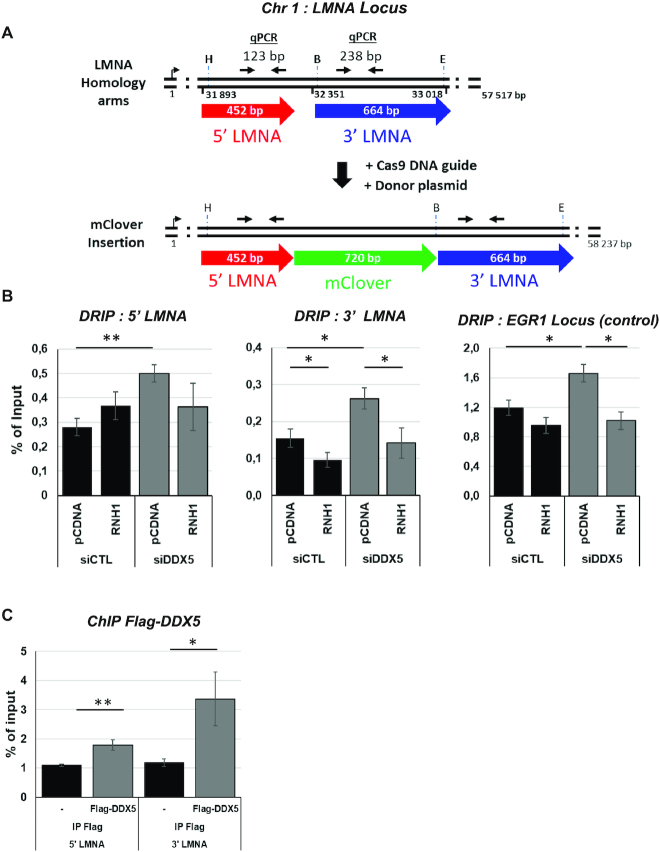
The CRISPR–Cas LMNA HDR system accumulates R-loops in a DDX5-dependent manner. (**A**) Illustration of the Cas9-directed knock-in of the Clover in the LMNA coding sequence. The red and the blue arrows represent both arms used for HDR. Cells were transfected with plasmids for CRISPR–Cas LMNA HDR analysis. B, E and H denote the location of the *Bsr*GI, *Eco*RI and *Hind*III restriction sites. qPCR amplification region is shown at the top of the red homology arm of the LMNA used for the DRIP-qPCR. (**B**) HEK293 cells were transfected with pcDNA or RNAse H1 (RNH1) expressing vector along with the siRNAs. Cells were then transfected with the CRISPR–Cas LMNA HDR system and iRFP plasmids and subjected to DRIP-qPCR analysis at both *LMNA* homology arms and the *EGR1* control locus. (**C**) HEK293 cells were transfected with Flag-DDX5 and subjected to ChIP-qPCR analysis. The bar graphs are the average and SEM from three independent experiments. Statistical significance was assessed using *t*-test: *P < 0.05 and **P < 0.01.

To examine whether the HDR defect observed in the Clover-LMNA system upon DDX5 depletion was a consequence of R-loop accumulation at the Clover-LMNA homology arm, we transfected the cells with V5-tagged RNAse H1 (Supplementary Figure S4A and B). RNAse H1 expression partially rescued the effect of DDX5 deficiency on HDR in both U2OS and HEK293 cells (Supplementary Figure S4A and B). To investigate whether DDX5 influenced DNA/RNA hybrids near the DNA cut site, we performed DRIP-qPCR using the DNA/RNA-specific S9.6 antibody. We assessed R-loops at the 5′ and 3′ homology arms within the digested fragment located at +31 893 and 32 351 bp, respectively, of the *LMNA* gene (Figure 8A). In siCTL cells, R-loop signals corresponding to ∼0.28% and ∼0.16% of the input were obtained for the 5′ and 3′ *LMNA* homology arms, respectively. In comparison, siDDX5 cells resulted in a significant increase in R-loops to ∼0.50% and ∼0.26% at the 5′ and 3′ *LMNA* homology arms, which corresponded to ∼2-fold overall increase (Figure 8B). Importantly, RNAse H1 expression prevented the increase in DNA/RNA hybrid in siDDX5 cells at *LMNA* (Figure 8B). The *EGR1* locus was used as a positive control for siDDX5 R-loop induction (Figure 8B) (17). In addition to the DRIP-qPCR, we also perform ChIP assays to confirm the presence of Flag-DDX5 at the 5′ and 3′ arms of the Clover-targeted site of the *LMNA* locus (Figure 8C). Indeed, Flag-DDX5 was enriched by ChIP at both the 5′ and 3′ regions of the *LMNA* locus (Figure 8C). Taken together, our findings suggest DDX5 localizes near DSBs to clear RNA from DNA/RNA hybrids to ensure HDR.

## DISCUSSION

The resolution of unscheduled R-loops is required to prevent DNA damage and maintain genomic stability (58,59). The regulatory complexes required to resolve this fundamental genetic structure near DSBs are not well understood. We previously reported that the DEAD box RNA helicase DDX5 plays an important role in resolving R-loops genome-wide (17,41). DDX5 deficiency led to accumulation of DNA/RNA hybrids at the DSBs in both the DART (17) and the CRISPR–Cas LMNA HDR analysis systems (Figure 7). In this study, we define a pivotal role for DDX5 in clearing the RNA moiety of R-loops near DNA breaks. DDX5-deficient U2OS cells exhibited asymmetric end deletions at DSBs overlapping a transcribed locus. We also observed DDX5 to be excluded from laser irradiation-induced DNA damage sites in an ATM/transcription-dependent manner, suggesting that it requires ATM or RNA for its expulsion from DSBs. DDX5-bound RNA transcripts of the DRGFP reporter gene in U2OS cells and I-SceI-mediated DSBs within the reporter significantly increased DDX5 binding and this was dependent on its ATP-dependent helicase activity. DDX5 deficiency delayed the recruitment of the single-strand DNA-binding protein RPA2 and EXO1 to laser irradiation-induced DNA damage sites resulting in HDR repair defects. These data suggest a model where chromatin-bound DDX5 captures R-loops and is displaced in a transcription- or ATM-dependent manner as soon as R-loops are resolved. The specific role for DDX5 in facilitating the clearance of RNA transcripts overlapping DSBs ensures proper DNA repair to maintain genomic stability.

We show that DDX5 is excluded from laser-induced DSBs in a transcription-dependent manner. Several other RBPs such as THRAP3 (60), hnRNPUL1 (61), RBMX (62), SAF-A and TLS/FUS (63) are excluded from laser-irradiated DNA damage sites. Like DDX5, the exclusion of many of these proteins from the DNA damage sites is transcription dependent. THRAP3 (60), like DDX5, does not have an initial recruitment phase to sites of DNA damage, unlike TLS/FUS, SAF-A (63), hnRNPUL1 (61) and RBMX (57). Our CLIP and ChIP data show that DDX5 is already present at sufficient amounts before the DSB. Once DNA damage is induced, CLIP shows stronger DDX5 RNA binding and ChIP shows less chromatin retention. These findings are consistent with the laser-induced DSB exclusion data suggesting that the DDX5/RNA complexes are evicted from DSBs.

Britton *et al.* showed that the recruitment and exclusion of SAF-A are two independent processes (63). For example, inhibition of PARP abolished SAF-A recruitment, but did not affect its exclusion (63). Thus, recruitment is not a prerequisite for exclusion. Similarly, we also found that treatment with PARP inhibitors had no effect on DDX5 exclusion from DNA damage sites (data not shown). As poly(ADP-ribosyl)ation (PARylation) is required for the recruitment of RBPs to DSBs, we treated cells with the inhibitors of PARG (which degrades PAR polymers and PARylation on target proteins) to enhance cellular PARylation level, and we found no recruitment of DDX5 at DSBs (data not shown). These findings further confirm that DNA damage-induced exclusion occurs from existing chromatin-bound DDX5 and not newly recruited DDX5 at DSBs.

Kinetic studies using the laser-induced DNA damage revealed that ATM inhibition did not affect the initial DDX5 exclusion, but caused a significant increase of DDX5 signal recovery at the breaks. These findings suggest that ATM activity is not required for initial phase of DDX5 exclusion, but it is required to regulate the return of DDX5 to chromatin after DNA damage.

Inhibition of ATM significantly reduced DDX5 exclusion from FokI-induced DSBs. It has been documented that DSBs induce in *cis* ATM-dependent transcription silencing near the breaks (24). The constant presence of DDX5 on chromatin before DSBs suggests that the exclusion observed using the FokI system is of the existing DDX5 bound to RNA before the cut by FokI. Once the DSB is induced by FokI, transcription is inhibited and the subsequent DDX5 recruitment is not occurring. Hence, net exclusion is visualized. In the presence of ATMi, the initial exclusion phase by DDX5 is likely occurring after the cut by FokI. However, after the DSB induced by FokI transcription is derepressed by ATMi, allowing DDX5 to return to the DSBs. Hence, no net exclusion is observed. These findings are consistent with DDX5 being excluded from sites in the presence of RNA. We were unable to find evidence that DDX5 is phosphorylated by ATM from PhosphoSitePlus^®^, proteomic studies (55) and our data (not shown). However, it is still possible that ATM phosphorylates DDX5 on sites not recognized by commercial phospho-SQ/TQ antibodies.

DDX5 associated with the RNA transcribed (encoding GFP fragment) at the DRGFP locus and I-SceI-induced DSBs caused significant increase of the reporter GFP RNA precipitated with DDX5 using the CLIP assay. These observations suggested that DDX5 resolves R-loops at DNA breaks. DSBs generally repress local gene transcription (24,64) and our RT-qPCR studies also showed a reduction of reporter gene expression when the cells expressed I-SceI. Thus, the increased amount of reporter RNA precipitated by DDX5 was unlikely a consequence of increased RNA expression. As only the soluble, but not the chromatin-bound DDX5 was precipitated in the CLIP experiments, the results suggest that I-SceI-cleaved DSBs might induce DDX5 dissociation from the breaks along with bound RNAs, consistent with the function of DDX5 in DNA/RNA hybrid resolution and exclusion from DSBs.

Remarkably, DDX5 deletion led to transcription-associated unscheduled DNA end deletions. The EJ5-GFP reporter system was designed to monitor end joining between two distal DSBs by monitoring the percentage of the GFP-positive cells when the two breaks mediated by I-SceI cleavage are ligated (56). In this study, we used it to identify end deletions near I-SceI site once *GFP* was excised. In siDDX5 cells, we observed frequent 5′-end deletions compared to the 3′-end deletions flanking the I-SceI cleavage site in the EJ5-GFP reporter system. This was consistent with the direction of the incoming transcription that is upstream of the I-SceI. Modification of the EJ5-GFP reporter system with inducible transcription unit further confirmed that DDX5 5′-end deletions required ongoing transcription. In siKu80 cells, we observed end deletions occurring on both sides of the DSB I-SceI site.

We also observed that DDX5-depleted cells displayed DNA HDR defects, as assessed by the CRISPR–Cas *LMNA* HDR assay (54). This defect was partially rescued by expression of RNAse H1 suggesting R-loops are forming in this reporter assay that hinders the HDR machinery. Furthermore, in siDDX5 cells, we detected at the *LMNA* locus increased R-loops by DRIP-qPCR. We conclude that DDX5 RNA helicase activity prevents the accumulation of R-loops that interfere with the HDR machinery. Recently, it was shown that the exosome subunit EXOSC10 resolves R-loops and clears RNA transcripts in DSB-flanking regions to facilitate HDR (65), suggesting there are many different machineries to clear RNA to ensure proper HDR.

In conclusion, our data suggest a new regulatory mechanism involving the RNA helicase DDX5 to regulate the resolution of R-loops to prevent DSB-associated DNA deletions and DNA recombination/repair defects. These observations are valuable from a therapeutic point of view, as DDX5 is overexpressed in breast cancer and acute myeloid leukemia (46,47). Furthermore, there is a DDX5 small molecule inhibitor RX-5902 (48) in clinical trial for triple-negative breast cancer. This is an actionable feature and readily suggests that a combination with other DNA damage-inducing drugs that increase number of DSBs would be promising strategy for late-stage cancers.

## Supplementary Material

zcaa028_Supplemental_File

## Notes

Present address: Jingwen Song, Donnelly Centre, University of Toronto, Toronto, Ontario M5S 3E1, Canada.
