# Supplementary material for: DDX5 resolves R-loops at DNA double-strand breaks to promote DNA repair and avoid chromosomal deletions
Source: NAR Cancer. 2020 Sep 25;2(3):zcaa028. doi: 10.1093/narcan/zcaa028 (PMC7520851; doi:10.1093/narcan/zcaa028)

Yu et al., Supplementary Figures

Figure S1. Effect of RNase H and ATM inhibitor on DDX5 recruitment at micro-irradiated DNA damage sites.

(*A*) U2OS cells were co-transfected with GFP-DDX5 and RNAseH1 (DDX5 + RNH1) or GFP-DDX5 alone (DDX5). (*B*) U2OS cells were untreated (NT) or treated with 10 μM of ATM inhibitor (ATM inhibition) for 4 hr. The cells were micro-irradiated and imaged every 20 seconds as described in the "Materials and Methods". The fluorescence intensity of GFP-DDX5 at DNA damage sites relative to an unirradiated area was quantified and plotted over time. Data show the mean relative fluorescence intensity ± S.E. of approximately 80 cells per condition from at least 3 independent experiments.

Figure S2. Effect of I-SceI cleavage on DRGFP reporter gene expression.

DRGFP cells were transfected with empty vector (- I-SceI) and the I-SceI-expressing vector (+ I-SceI) respectively, and the expression of the reporter gene was analyzed by RT-qPCR using the same primers as shown in Figure 4. *GAPDH* expression was used for normalization. The graph shows the average and standard error of the mean (SEM) from three independent experiments. Statistical significance was assessed using Student's t-test; *, $p < 0.05$.

Figure S3. DDX5 deficiency leads to cellular hypersensitivity to etoposide treatment.

(A) Illustration of FACS-based cell survival analysis. Wild type U2OS cells were transfected with control siRNA and U2OS stably expressing GFP were transfected with siRNAs targeting specific genes as indicated. The cells were then harvested two days after transfection and mixed

in an approximately 1:1 ratio. These cells were plated and exposed to etoposide with higher dose (2 μM) for 2 hr or lower dose (0.4 μM) for 24 hr. *(B)* FACS analysis was performed following seven to ten days of recovery. *(C)* Data are plotted as a ratio of GFP+ to GFP- cells normalized to untreated control. The graphs show the average and standard error of the mean (SEM) from four independent experiments. Statistical significance was assessed using Student's t-test; ***, $p < 0.001$.

Figure S4. CRISPR-LMNA HDR is partially rescued by RNH1 expression.

Twelve hours after transfection with siCTL or siDDX5, U2OS *(A)* and HEK293 *(B)* cells were transfected with pcDNA or RNAseH1 (RNH1) expressing vectors. Then, cells were transfected with the CRISPR-Cas LMNA HDR system and iRFP plasmids. Forty eight hours after, cells were fixed and subjected to immunofluorescence against V5 tag. Clover positive cells among iRFP and V5 positive cells was quantified and normalized to siCTL condition. The experiment was performed 3 times, with at least 150 cells counted for each replicate. Statistical significance was assessed using Student's t-test. *, $p < 0.05$ and **, $p < 0.01$.

Yu et al., Figure S1

A

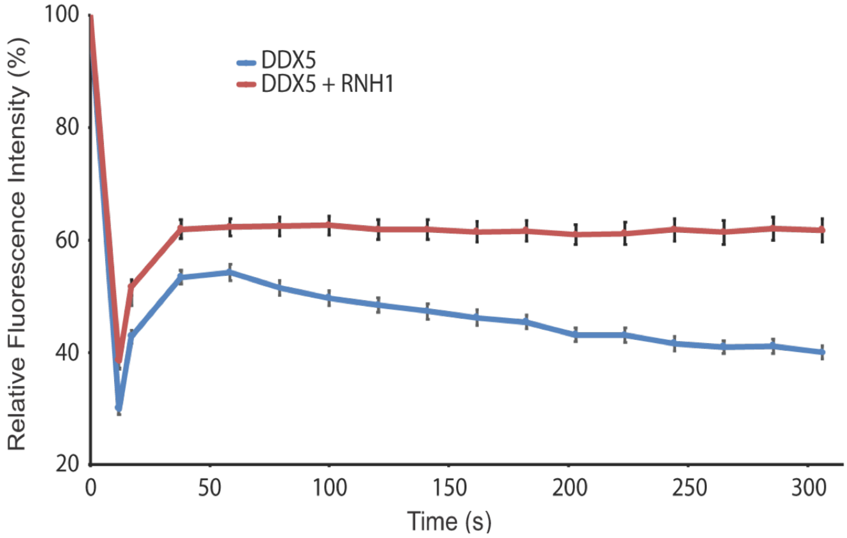

B

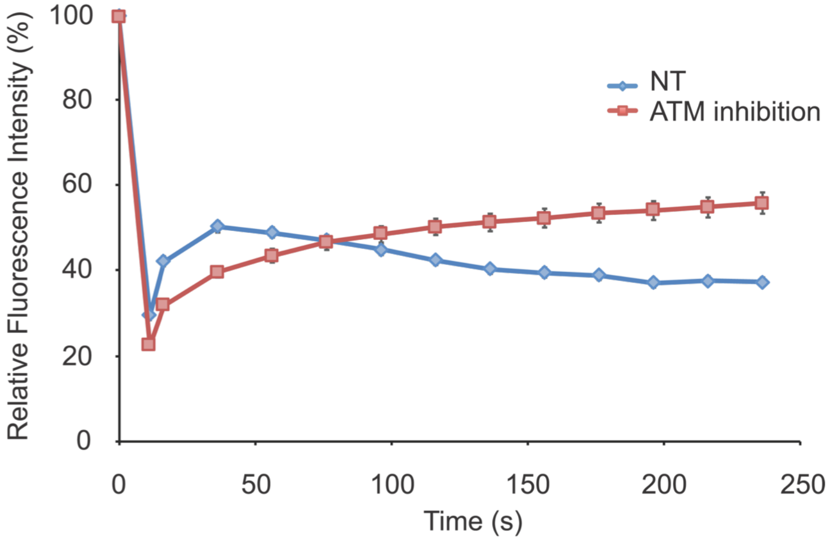

Yu et al., Figure S2

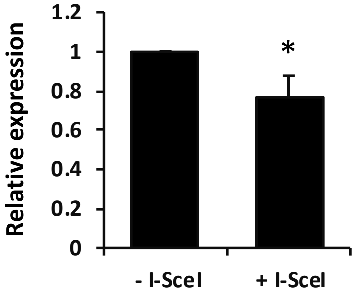

Yu et al., Figure S3

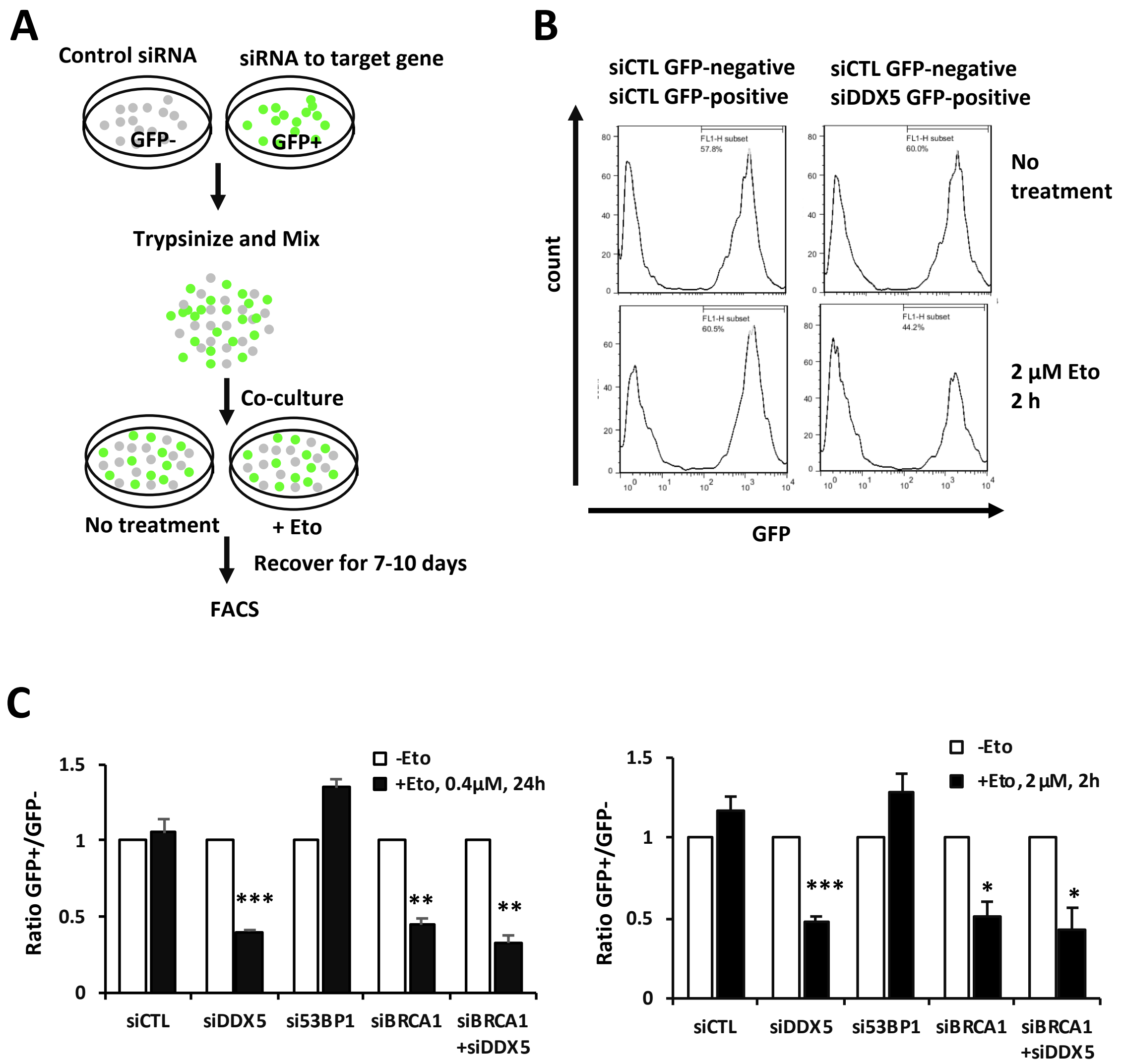

Yu et al., Figure S4

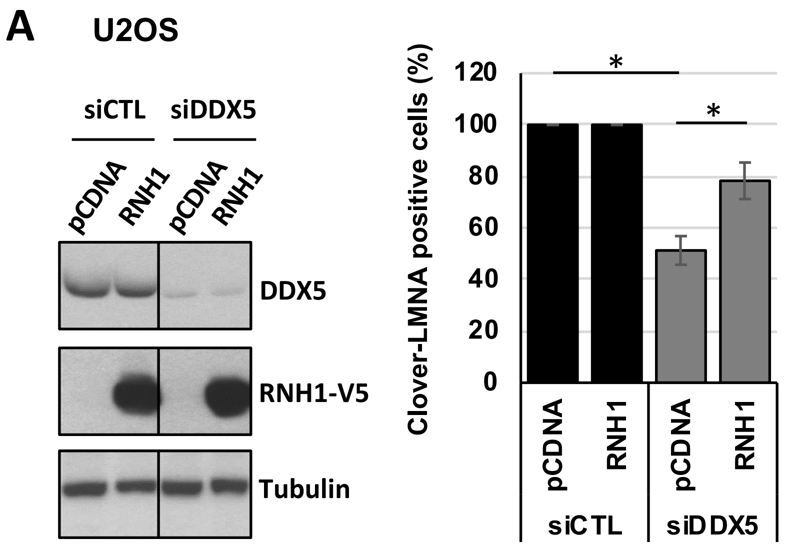

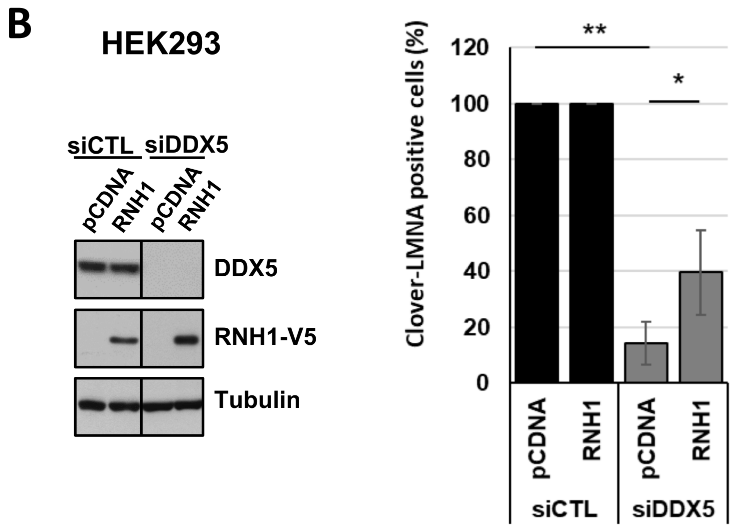

Supplement: zcaa028_Supplemental_File [file zcaa028_supplemental_file.pdf]
